# Editorial Chit Chat

**Published:** 1870-10

**Authors:** 


### EDITORIAL CHIT CHAT.

ASKED FOR.—The article on "How to Choose a Doctor," by Rev. T. K. Beecher, was written for and published in the BISTOURY, in 1868.

PROF. VON GRAEFE, the world-renowned oculist of Berlin, is dead. He was the originator of several important operations upon the eye, which gave him a world-wide reputation.

CAIN WANTED.—Sam Taber, Agricultural Editor of the *Advertiser* wants somebody to give him a cain. Why in fury don't you *raise* one, Sam?

PERSONAL.—Mr. A. L. Williams, Canandaigua's popular jeweler and nimrod, is expected in this city soon, to spend two months with his friends here. Advice to the Game.—"Lay low!"

QUEER.—The Foreman in the *Gazette* office at Troy, Pa., has a queer way of "making up" his paper. He places his BIRTHS before his MARRIAGES. That isn't the way things are done up this way—we're a more moral people than that.

QUERY.—Can a man, afflicted with Catarrh, so common just now, expect-to-rate with a fashionable young lady, in her parlor? If so, why? For the successful solution of this problem we will forward one of Mark Twain's new war maps.

CITY MARKET.—We see the City Fathers have ordered the opening of a market place for the sale of country produce. This is as it should be. Hope we may soon have our regular market days, so that we may know when to expect farmers with their vegetables, &c.

DR. PAUL SCHOEPPE—the Doctor, who was convicted last winter of the murder of Miss Stennecke, in Pennsylvania, asks the governor to discharge him in order that he may visit his father-land and fight that "impudent man, Napoleon."

"UNCLE BOB."—We have received several communications from subscribers expressing their appreciation of our contributor's articles upon children and their management. Some have asked us whether Mr. Beecher was not the Author of the articles alluded to. We presume "Uncle Bob" has no objection to being known by his real name, so that our readers may credit his articles to Mr. ROBERT A. HALL.

THE WAR.—The American ladies now resident in Paris, have formed a Sanitary Commission, similar to the one which did so much for the comfort of the sick and wounded, during our own war. Mrs. Burlingame is President of the Society, and Mrs. S. W. Evans, wife of the celebrated dentist, is Vice-President. Telegraphic reports say they are doing heroic work in the Hospitals of Paris and on the field.

CHILDLESS WOMEN IN NEW YORK.—Dr. Silas Loomis, of Washington, writes to the *National Med. Jour.* that twenty-four per cent. of the married women of this State are childless, and nearly twenty per cent. are incompetent after bearing one child.

Here is a text for ministers to preach from. If they would only preach more upon the sin of abortion, and if physicians would instruct the people more upon the danger of this crime to health and happiness, it would have a salutary effect upon the morals and healthfulness of the State.

RETURNED TO JOURNALISM.—We learn that Geo. S. Brown, Esq., a popular journalist of the West has purchased an interest in the Toledo *Democrat* and is one of the Editorial Corps. We congratulate the *Democrat* and its readers upon securing the services of so excellent a writer as Mr. Brown.

---

J. A. REDFIELD, ESQ., the popular Superintendent of the Northern Central Rail Road, has been quite ill for several weeks, and confined to his bed. We are glad to see him up again however, and hope he may soon be able to take a ride over the new extension of his road at Ralston. When that occurs, it will doubtless be a *plain* case of convalescence.

---

TO BE CONGRATULATED.—The citizens of Scranton, Pa., upon the advent in their midst of those two accomplished gentlemen, Messrs. Robert A. Hall and Ben H. Pratt, who have gone into the Book business at No. 414 Lackawana Avenue. Messrs. Hall & Pratt were two of our most respected and popular citizens, who leave hosts of friends here, to wish them success in the city of their adoption.

---

NEW MEDICAL JOURNAL.—We have just received the first number of a new medical journal, published by J. B. Lippincott & Co. at Phliadelphia. It is printed on elegant tinted paper, with clear type and is a model of typographical neatness. It is to be the recognized organ of the Phila. Med. Society, and has for contributors *all* of the best Physicians and Surgeons of that City. The list of contributors is headed by such able and celebrated men as Gross. Pancoast, Da Costa, Austin Flint, Meigs, etc., etc. It is a semi-monthly journal, called the MEDICAL TIMES, and the terms are four dollars per annum.

---

THE NEW ROUTE.—It will be observed that we publish the time table of the Lehigh Valley Rail Road, the new route to New York City and other Eastern Cities. To those persons who desire to escape the frequent occurring accidents of the Erie, the Lehigh Valley Route offers not only a safe, but also a highly picturesque ride through the renowned Susquehanna, Wyoming and Lehigh Valleys. Ask for tickets *via* the Lehigh Valley Road. It is safer, shorter, and a more pleasant and enjoyable route to the East.

AT IT AGAIN.—The irrepressible Cadieux, the patriot, orator, statesman, author, doctor, wife-beater and *dam-phool* generally, has turned up in Syracuse, as editor of a French paper, called the *Le Citoyen Americain.* Ca-dux is an egregious humbug, and unless the people of Syracuse keep a good look out, they will have a first class revolution organized in their midst, with Cadieux as self proclaimed Emperor.—*Vive la Ca-dux !*

---

CITY HOSPITAL.—The little, free hospital opened by us several months ago, for the treatment of the indigent sick, is now full. This fact exhibits the necessity for suitable hospital buildings for the accommodation of the very many poor people in our midst, and those who chance to be thrown among us, sick and injured. We hope some provision will be made by our well-to-do citizens, for the care and comfort of these poor people. Such an institution is of vastly more importance than some other benevolent enterprises that are supported through the benevolence of our citizens.

---

NOT RELIABLE.—The West Bloomfield correspondent of the *Advertiser* is not reliable. He's pretty HENDEE with a pen, but then he has a way of exaggerating affairs that is unpleasant. He informs the world, through the *Advertiser*, that we only killed four of the thirty-six squirrels which we recently shipped home from there, to our friends in Elmira; when HAMLIN is ready to testify that we killed FIVE of them, (counting the fox's tail, and which he insinuates was killed the day before). Now, MARV, we don't want you to PECK our story to pieces in that manner again —it places a halo of doubtfulness about our assertions, that does not at all harmonize with our reputation for veracity.

---

TO LEAVE US.—Mr. SAMUEL CONKEY, the Sculptor, is about to leave us and take up his residence in Chicago. This step is rendered necessary from the fact that our city is not yet large enough, or her citizens do not appreciate Art sufficiently, to keep a first class and talented Artist among us. Mr. Conkey has executed several most excellent busts, in marble, and has made numerous ideal works, that have been exhibited in the New York Galleries of Art, calling forth numerous and highly complimentary criticisms from the City papers, which have given him rank among the very best Sculptors of the United States. We recommend him to the Chicago people as being a gentleman, in every sense of the word, and a Sculptor of rare skill and industry.

We also learn that our best landscape and portrait painter is soon to follow Mr. Conkey to his new, western home. It seems Mr. WATERS has also outgrown our city and must seek a home in a larger one, where he can secure prices that will be remunerative for the very excellent paintings that he is now engaged upon. We are sorry to lose two such pleasant gentlemen and skillful Artists. It will be many a day before Elmira can boast of a pair equal to them.

---

OUR PUBLIC SCHOOLS.—If there is any one thing more than another that entitles Elmira to eminence among the cities of New York, it is the admirable manner in which her public schools are conducted. We have had the pleasure of visiting several institutions of learning in this State, and have conversed with many teachers and others from abroad, and all agree that Elmira stands far in advance of any city in the State, in point of excellence, in the management of her graded, public schools. The new School House in the 5th Ward, now nearly completed, is a model of architectural design and beauty. It is by far the handsomest in the city, and reflects great credit upon our Board of Education for furnishing so beautiful a building and so healthy a locality for the education of our children. We are sure the school will be one of the very best in the State, under the able management of Mr. Parley Coburn, and we congratulate that gentleman upon his prospective "change of base."

---

DISTINGUISHED PHYSICIAN DEAD.—SIR JAS. Y. SIMPSON, of Edinburgh, is dead. He was one of the most distinguished physicians of Europe, and was the discoverer of the anæsthetic properties of chloroform. He was born in 1811—received his education at the University of Edinburgh, where he took the degree of M. D. in 1832. In 1842 he was appointed Professor of Midwifery. In 1847 he first produced insensibility during accouchment by the use of sulphuric ether, and soon after employed chloroform for the same purpose. In 1849 he was elected President of the Edinburgh Royal College of Physicians. He enjoyed the largest private practice of any physician in Europe. In 1866 he was created a Baronet, and in the same year received the honorary degree of D. C. L.

WOMEN NURSES.—The Grand Duchess of Baden has organized a corps of nurses for the Prussian Army. She requires that all who desire to join her army shall be clothed in plain dresses and caps. She complains that recruiting is not lively, as the pretty girls will not render themselves homely in plain caps, and the homely girls refuse to render themselves more so by throwing off their curls and chignons. The uniform adopted by the Grand Duchess, for her nurses, is a plain, dark blue, linen dress, with white collar, sleeves loose, wide and buttoned at the wrists. Those who belong to the hospital, or whose duty it is to nurse the sick, wear a small bow of blue, narrow, satin riband, with "Frauen-Verein King, 1870," printed thereon, and is worn on the left side. Those who act in the capacity of cooks, wear a green bow, and those who attend the linen department wear a white one.

The hospital bears a white flag with a red cross, while the regular hospital nurses, men and women, wear a white band with red cross around the left arm.

---

LAKE ELDRIGE AND THE DOCTOR.—Lake Eldridge is rapidly becoming a little paradise. It is a real pleasure to drive there in the evening, and observe the many handsome walks, drives, fountains, statues, flowers and ponds, which the bountiful generosity of the Doctor has placed at the disposal of the citizens of Elmira. Almost imperceptibly has this romantic retreat sprung into loveliness and grandeur under the magic influence of Dr. Eldrige's taste and money. Every pleasant Sunday, hundreds of people, rich and poor, from the humblest to the most exalted, may be seen driving, sitting and walking in the cool and pleasant retreats afforded them by his munificence. Upon every hand we hear words of praise for one who has done so much for the enjoyment of others. We propose that some more substantial token of our apprec'ation be awarde 1 Dr. Eldridge for his wonderful generosity and lavish expenditure of money for the enjoyment of his fellow citizens. Let a subscription be opened, in which everybody, rich and poor, shall be allowed to contribute such sums as they can afford, for the purchase of a suitable testimonial, from the entire community, to Dr. Eldrige. We would suggest the erection of a life size, marble statue of the Doctor himself, upon the lake grounds, to be executed by our own sculptor, Conkey.

Such an act would be worthy of the city and of the man who has done so much for her and her citizens. We hope some responsible and energetic person will start such a subscription and give our people an opportunity to express their appreciation of the Doctor's efforts to beautify our city,and for affording amusement and recreation for our people. Call on us for our share.

---

A SURGICAL OPERATION.—We anticipate that the next number of the *Bistoury* will give to the public the particulars of one of the most remarkable and successful surgical operations ever performed in the States. The operation was performed in the Fifth Ward, of this city, on Saturday last, and not only the surgeon who performed it but also his assistant who aided him, at once took *rank* with the most eminent men in or out of the profession. The operation was performed upon the Memphitis Americana, a member of the digitgrade family, for the purpose of removing two glands containing a liquid possessing valuable medicinal powers, and as above indicated, was eminently successful and entirely satisfactory. Indeed, we understand that both surgeon and his assistant were *more than satisfied* with the result of the operation, while the patient is reported to be in a fair way of recovery, both from the effects of the chloroform and of the surgical operation. Should the case not be reported in the *Bistoury*, full particulars in regard to it can be obtained by inquiring of or addressing Dr. T. S. UPDEGRAFF, or one of our most popular book sellers, of *high standing*, residing in the Fifth Ward.—*Daily Advertiser.*

Now, Sam, that's unfair. You did not give any credit to a scientific gentleman and physician, who was present at the operation, and who, by his attainments as a Naturalist, rendered valuable aid in *discovering* and removing the glands above mentioned. We do not know whether the bird (?) operated upon will be described in his forthcoming ornithological work —"The Birds of New York," but certain it is that the medicine obtained in the operation referred to, can be had at *wholesale* by calling at his jobbing drug house, corner of Lake and Water Sts., where you can also hear the Doctor's favorite latin exclamation, as delivered at the conclusion of the operation above referred to, viz :—*Iti sapiss potitis !*

